# Surgery Matters: Progress in Surgical Management of Gastric Cancer

**DOI:** 10.1007/s11864-022-01042-3

**Published:** 2023-01-19

**Authors:** Katharina Beyer

**Affiliations:** Department of General and Visceral Surgery, Charité University Medicine Berlin, Campus Benjamin Franklin, Hindenburgdamm 30, 12203 Berlin, Germany

**Keywords:** Gastric cancer, Gastrectomy, D2 lymphadenectomy, Function-preserving gastrectomy

## Abstract

The surgical treatment of gastric carcinoma has progressed significantly in the past few decades. A major milestone was the establishment of multimodal therapies for locally advanced tumours. Improvements in the technique of endoscopic resection have supplanted surgery in the early stages of many cases of gastric cancer. In cases in which an endoscopic resection is not possible, surgical limited resection procedures for the early stages of carcinoma are an equal alternative to gastrectomy in the field of oncology. Proximal gastrectomy is extensively discussed in this context. Whether proximal gastrectomy leads to a better quality of life and better nutritional well-being than total gastrectomy depends on the reconstruction chosen. The outcome cannot be conclusively assessed at present. For locally advanced stages, total or subtotal gastrectomy with D2 lymphadenectomy is now the global standard. A subtotal gastrectomy requires sufficiently long tumour-free proximal resection margins. Recent data indicate that proximal margins of at least 3 cm for tumours with an expansive growth pattern and at least 5 cm for those with an infiltrative growth pattern are sufficient. The most frequently performed reconstruction worldwide following gastrectomy is the Roux-en-Y reconstruction. However, there is evidence that pouch reconstruction is superior in terms of quality of life and nutritional well-being. Oncological gastric surgery is increasingly being performed laparoscopically. The safety and oncological equivalency were first demonstrated for early carcinomas and then for locally advanced tumours, by cohort studies and RCTs. Some studies suggest that laparoscopic procedures may be advantageous in early postoperative recovery. Robotic gastrectomy is also increasing in use. Preliminary results suggest that robotic gastrectomy may have added value in lymphadenectomy and in the early postoperative course. However, further studies are needed to substantiate these results. There is an ongoing debate about the best treatment option for gastric cancer with oligometastatic disease. Preliminary results indicate that certain patient groups could benefit from resection of the primary tumour and metastases following chemotherapy. However, the exact conditions in which patients may benefit have yet to be confirmed by ongoing trials.

## Introduction

According to GLOBOCAN estimates, gastric cancer is the fifth most common type of cancer in the world. In addition, it was the fourth largest cause of cancer-related death in 2020 [1]. A population-based cohort study conducted in Sweden revealed an unchanged survival rate for non-cardiac cancer in the period from 1990 to 2015. The survival of the group of patients who underwent surgery improved significantly [2]. Improved patient selection, centralisation of gastric cancer procedures, interdisciplinary therapy planning and the increased use of multimodal therapy may explain the improved survival following surgery.

While the establishment of perioperative chemotherapy represents a milestone in improving oncological results in locally advanced gastric cancer in the Western world [3], the further development and standardisation of surgical techniques is also essential.

This review will summarize how advances in surgical therapy have improved oncological outcomes, perioperative morbidity and quality of life.

## Early gastric cancer

### Endoscopic therapy

Prerequisites for endoscopic therapy include suitability for an en bloc resection and a negligible risk of lymph node metastasis. According to the Japanese gastric cancer treatment guidelines from 2018, an endoscopic resection is a standard procedure (absolute indication) when the risk of harbouring lymph node metastasis is lower than 1% [4] and when the treatment effect of endoscopic resection is expected to be equivalent to that of surgical resection. An investigational (expanded) treatment is given when the risk of lymph node metastasis is lower than 1% but the oncological equivalence of endoscopic treatment and surgery has not been proven [4].

Along with oncological equivalence, technical feasibility is a prerequisite for endoscopic treatment. When endoscopic mucosal resection (EMR) was performed, the indications for endoscopic therapy were limited to mucosal carcinomas less than 2 cm in size without ulceration with a grading of G1/2. The development of endoscopic techniques that allow en bloc resections of larger lesions has enabled the extension of endoscopic therapy in the past few years. Japanese guidelines consider two additional conditions absolute indications that endoscopic submucosal dissection is appropriate. The first condition is a differentiated-type adenocarcinoma without ulcerative findings, in which the depth of invasion is clinically diagnosed as T1a and the diameter is > 2 cm. The second condition is a differentiated-type adenocarcinoma with ulcerative findings (UL1), in which the depth of invasion is clinically diagnosed as T1a and the diameter is ≤ 3 cm. Gotoda et al. suggested that the risk in these conditions is negligible because they found no lymph node metastases in these cases [5].

There have been studies comparing the survival after endoscopic submucosal dissection (EDS) of patients who met guideline criteria to patients who met the extended criteria. Many studies from Asia have demonstrated equal outcomes for both types of patients. Nevertheless, it was generally more feasible to perform R0 resections in patients from the group that followed the guideline criteria [6–8].

There are often concerns about the transferability of data to other countries. However, there are data from the West revealing that ESD is adequate when using the expanded criteria [9]. For this reason, the European guidelines recommend endoscopic resection for patients within the guideline criteria. For patients who meet less than two parameters of the extended criteria, the guidelines recommend considering endoscopic resection using ESD [10].

There is not yet a consensus on the oncological equivalence of endoscopic therapy compared to surgery in treating undifferentiated-type mucosal cancer. The consensus is that undifferentiated-type mucosal lesions > 2 cm must be treated surgically. However, the surgical treatment of undifferentiated-type mucosal lesions smaller than 2 cm has not been thoroughly investigated. The concern about endoscopic treatment of these smaller lesions is due to lymph node metastasis. Whereas Gotoda reported a 0% rate for lymph node metastasis in undifferentiated gastric cancer < 2 cm without ulceration, there are other trials showing metastasis rates of up to 5% [11]. The JCOG 1009/1010 trial investigated the efficacy and safety of endoscopic submucosal dissection for undifferentiated-type mucosa gastric cancer less than 2 cm in diameter without ulceration. The study revealed en bloc resections of 99% and a curative resection rate of 71% with a 5-year overall survival (OS) of 99.3% [12].

### Function-preserving gastrectomy for early gastric cancer

For early gastric carcinomas, in which endoscopic resection is not feasible for some reason, surgery is recommended. While gastrectomy with D2 lymphadenectomy is the global standard in the surgical treatment of gastric cancer, it is unclear whether limited function-preserving resection procedures would also be sufficient for T1N0 carcinomas. The evidence for these procedures comes almost exclusively from Asia.

The Asian guidelines consider a function-preserving gastrectomy with a D1+ lymphadenectomy to be sufficient insofar as there is no suspicion of lymph node metastases [4, 10]. The German guidelines recommend a total or subtotal gastrectomy with D2 lymphadenectomy in such cases [13]. For the T1 stage, the European guidelines state that these patients may require less radical surgery than more advanced stages and that a D1+ lymphadenectomy may be sufficient [10]. However, function-preserving gastrectomy is not further detailed in this guideline, which reflects the low incidence of these stages in Europe.

The Japanese guidelines consider a D1 lymphadenectomy sufficient for T1a tumours that do not meet the criteria for ESD and for differentiated-type T1bN0 tumours that are smaller than 2.5 cm in diameter [4]. A D1+ lymphadenectomy is recommended for more advanced T1N0 tumours (Table 3).

The most frequently performed function-sparing resection procedures in the world are proximal gastrectomy for carcinoma of the upper stomach and pylorus-sparing gastrectomy for carcinoma of the middle third of the stomach. These procedures have the potential to lessen the impairments caused by post-gastrectomy syndromes. However, it must be borne in mind that it is unclear to what extent these recommendations can be transferred to the Western world.

### Proximal gastrectomy

#### Oncological outcomes

In a proximal gastrectomy, the cardia and upper portions of the gastric corpus are resected; more than half of the stomach remains. The extent of an appropriate lymphadenectomy in a function-preserving gastrectomy has been defined by the Japanese guidelines; the D1 lymphadenectomy stations for a proximal gastrectomy include 1, 2, 3a, 4sa, 4sb, and 7 (Table 1, Table 2). If a D1+ lymphadenectomy is performed, stations 8a, 9, and 11p must also be removed [4]. For tumours that affect the oesophagus, the lymphadenectomy also includes station 110 (Table 2). Due to the lower extent of the D1+ lymphadenectomy in proximal gastrectomy, the vagal branches to the liver and pylorus can be preserved.

**Table 1:** Numbering of lymph node stations according to the current classification of the Japanese Research Society for Gastric Cancer. Reprinted with permission from: Japanese Gastric Cancer Association, Japanese Classification of Gastric Carcinoma: 3rd English edition, Gastric Cancer (2011) 14: 101-112

**Station**	**Lymph nodes**
1	Right paracardial nodes
2	Left paracardial nodes
3	Nodes along the lesser curvature
4	Nodes along the greater curvature
	4sa: along short gastrics
	4sb: left greater curvature lymph nodes along the left gastroepiploic artery
	4d: right greater curvature lymph nodes
5	Suprapyloric lymph nodes
6	Infrapyloric lymph nodes
7	Nodes along the left gastric artery
8	Nodes along the common hepatic artery
9	Nodes around the celiac axis
10	Splenic hilar nodes
11	Nodes along the splenic artery
12	Nodes in the hepatoduodenal ligament, in the caudal half between the confluence of the right and left hepatic ducts and the upper border of the pancreas
	12a: Nodes along the proper hepatic artery
	12b: Nodes along the bile duct
	12p: Nodes along the portal vein
13	Lymph nodes at the posterior aspect of the pancreatic head
14	Lymph nodes at the root of the mesentery
	14v: Nodes along superior mesenteric vein
	14a: Nodes along superior mesenteric artery
15	Lymph nodes in the mesocolon of the colon transversum
16	Para-aortic lymph nodes
17	Lymph nodes on the anterior surface of the pancreatic head
18	Lymph nodes along the inferior border of the pancreatic body
19	Infradiaphragmatic lymph nodes predominantly along the subphrenic artery
20	Hiatal paraesophageal lymph nodes
110	Paraesophageal lymph nodes in the lower mediastinum
111	Supradiaphragmatic lymph nodes distant from the oesophagus
112	Posterior mediastinal lymph nodes distant from the oesophagus and the hiatus

**Table 2:** Lymph node dissection in gastrectomy for gastric cancer [4]

**Type of gastrectomy**	Extend of lymphadenectomy	Lymph node stations
Total gastrectomy	D1	No. 1–7
	D1+	D1 + No. 8a, 9, 11p + No. 110 for tumours invading the oesophagus
	D2	D1 + No. 8a, 9, 11p, 11d, 12a + No. 19, 20, 110, 111 for tumours invading the oesophagus
Distal gastrectomy	D1	No. 1, 3, 4sb, 4d, 5, 6, 7
	D1+	D1 + No. 8a, 9
	D2	D1 + No. 8a, 9, 11p, 12a
Pylorus-preserving gastrectomy	D1	No. 1, 3, 4sb, 4d, 6, 7
	D1+	D1 + No. 8a, 9
Proximal gastrectomy	D1	No. 1, 2, 3a, 4sa, 4sb, 7
	D1+	D1 + No. 8a, 9, 11p + No. 110 for tumours invading the oesophagus

The concept of less extensive lymphadenectomy for proximal gastric cancer was also supported by data from patients diagnosed with more advanced tumours. A retrospective analysis of 202 patients diagnosed with T2/T3 tumours limited to the proximal stomach did not demonstrate a single lymph node metastasis in the supra- or infrapyloric lymph nodes. The lymph nodes along the right gastroepiploic artery and the proper hepatic artery were involved in < 1% of cases [14].

There are numerous retrospective studies and meta-analyses showing no difference in the overall 5-year survival between T1 tumour patients who underwent a proximal gastrectomy and T1 tumour patients who underwent a total gastrectomy [15–19]. A Japanese prospective non-randomised study and a Chinese prospective randomised study have additionally confirmed the oncological equivalence of both methods for early gastric cancer [20, 21].

Previous literature indicates that a proximal gastrectomy with a D1+ lymphadenectomy represents an oncologically adequate operation for proximal gastric carcinomas, particularly for T1 carcinomas, without evidence of lymph node metastases. There is retrospective data that reveals significantly higher recurrence rates in cases of more advanced carcinomas [22]. The available data may vary in usefulness due to the predominantly retrospective nature of the studies.

#### Functional outcomes following proximal gastrectomy

One potential benefit of the proximal gastrectomy is that nutritional status may deteriorate less compared to the total gastrectomy [20, 23]. However, this advantage is accompanied by higher reflux rates and anastomotic stenosis [19, 20, 24]. The exact extent of the reflux symptoms depends on which reconstruction is chosen.

There are several potential options for reconstruction after proximal gastrectomy. The commonly chosen options are oesophagogastrostomy, jejunal interposition, and double-tract reconstruction. Double-tract reconstruction has become more common in the past few years.

Oesophagogastrostomy is the simplest method and offers the advantage of a physiological reconstruction. The method has a low risk of technical failure since only one anastomosis is necessary. Another advantage is the straightforward surveillance of the gastric remnant due to the direct endoscopic access from the oesophagus. A propensity score-matched analysis showed better nutritional status after proximal gastrectomy compared to total gastrectomy, but the benefit was offset by higher rates of reflux symptoms and anastomotic stenosis [25].

A method to improve reflux symptoms after a proximal gastrectomy is to form a tube from the residual stomach. A Chinese prospective cohort study, which included 66 consecutive patients, indicated that the formation of a gastric tube led to a reduction in reflux symptoms and in endoscopically confirmed reflux esophagitis [26].

In addition to gastric tube reconstruction, anti-reflux procedures that can be used in addition to oesophagogastrostomy have been developed. The double-flap reconstruction is an anti-reflux measure that is frequently implemented in Asian countries. During the double-flap reconstruction, an H-shaped seromuscular flap is formed from the anterior wall of the residual stomach, which is placed around the oesophagogastrostomy after suturing the anastomosis [27]. Retrospective studies and a recent meta-analysis highlighted an improvement in reflux oesophagitis rates, which decreased from an average of 19 to 9% by adding the double-flap reconstruction to the oesophagogastrostomy [24, 28, 29].

Another measure to minimise postoperative reflux is the side overlap oesophagogastrostomy with fundoplication by Yamashita (SOFY) method; the initial results from this method were presented in 2017 [30]. In this procedure, a linear stapler is used to create a slit-shaped anastomosis between the right side of the oesophagus and the anterior wall of the residual stomach. The oesophagus, stomach and diaphragm are then fixed against each other with sutures. This construction serves as a backflow prevention mechanism and has been modified since its introduction [30]. Preliminary results revealed reflux rates of 2.8% when applying the modified SOFY method [31]. However, studies with high case numbers are not available for this method.

Another strategy to avoid postoperative reflux is the creation of an oesophagojejunostomy. Jejunal interposition, jejunal pouch interposition and double-tract reconstruction are procedures that can be used for this purpose.

Jejunal interposition consists of an 8–15 cm long pedicled loop of proximal jejunum being interposed between the oesophagus and the gastric remnant. The jejunal interposition serves as a substitute for the gastroesophageal junction by preventing reflux [32]. When compared to oesophagogastrostomy, a meta-analysis of six studies demonstrated that jejunal interposition has the disadvantages of inconvenient operating time, intraoperative blood loss and a longer hospital stay. These effects demonstrate the relatively more convenient reconstruction in oesophagogastrostomy. However, there was a trend towards better reflux control in the jejunal interposition group [33].

An additional retrospective study with 301 patients examined the functional outcome after jejunal interposition compared to reconstruction with a gastric tube. Although the operation time for jejunal interposition is longer, there were fewer cases of reflux esophagitis and fewer post-gastrectomy syndromes [34].

However, residual food is a prevalent issue in jejunal interposition. It becomes even more prevalent when a jejunal pouch is used [24].

To overcome the high technical requirements for jejunal interposition, double-tract reconstruction was developed. When laparoscopic techniques are used, double-tract reconstruction is less prone to technical failure. During double-tract reconstruction, the jejunum is divided approximately 15-cm distal from the ligament of Treitz. The distal limb is anastomosed to the oesophagus. A side-to-side jejunogastrostomy is performed 15 cm below the oesophagojejunostomy. A jejunogastrostomy is performed approximately 40 cm below the oesophagojejunostomy. A meta-analysis of seven retrospective studies comparing double-tract reconstruction to total gastrectomy demonstrated that double-tract reconstruction is superior in maintaining adequate B12 levels. This superior effect does not come at the expense of reflux or anastomotic strictures [35]. In addition, an analysis of the postoperative outcomes of nine studies revealed low rates of reflux oesophagitis (9.6%) following double-tract reconstruction. In that study, remnants of food also caused an issue in the reconstruction [24]. The Korean KLASS05 study randomised laparoscopic proximal gastrectomy with double-tract reconstruction versus laparoscopic total gastrectomy. The short-term results of this study indicate no disadvantage of a proximal resection in terms of perioperative morbidity [36]. The long-term results regarding the nutritional status and the oncological outcomes are still pending.

### Pylorus-preserving gastrectomy

Pylorus-preserving gastrectomy is a limited resection procedure that can be useful for early carcinomas in the middle third of the stomach. The current Japanese guidelines consider it as an appropriate method to treat cT1N0 tumours, with the distal border of the tumour being at least 4 cm from the pylorus [4] (Table 3). A D1 lymphadenectomy in a pylorus-preserving gastrectomy includes the stations no. 1, 3, 4sb, 4d, 6, and 7. In the case of a D1+ lymphadenectomy, the stations no. 8a and 9 must also be removed (Table 2). Leaving the lymph nodes adjacent to the right gastric artery requires a thorough consideration of the risk of metastases in the lymph node at station no. 5. Skeletonisation of the infrapyloric artery also carries the risk of the incomplete dissection of station no. 6 [37].

**Table 3:** Conditions under which a function-preserving gastrectomy can be considered according to Asian guidelines. A prerequisite for surgical therapy of early gastric cancer is that the tumour does not meet the criteria for endoscopic submucosal dissection (ESD)

		**Chinese guidelines (CSCO 2021)**	**Japanese guidelines (JGCA 2021)**	**Korean guidelines (KGCA 2018)**
**Proximal gastrectomy with D1 LAD**	Tumour stage	cT1a cN0	cT1a cN0	-
		cT1b cN0 (differentiated type, lesion size < 1.5 cm)	cT1b cN0 (differentiated type, lesion size < 1.5 cm)	
	Tumour localisation	Proximal side	Proximal tumours where more than half of the distal stomach can be preserved.	-
**Proximal gastrectomy with D1+ LAD**	Tumour stage	cT1b cN0 tumours that do not meet the criteria for D1 LAD	cT1b cN0 tumours that do not meet the criteria for D1 LAD	cT1 cN0
	Tumour localisation	Proximal side	Proximal tumours where more than half of the distal stomach can be preserved	Upper third of the stomach
**Pylorus-preserving gastrectomy with D1 LAD**	Tumour stage	cT1a cN0	cT1a cN0	-
		cT1b cN0 (differentiated type, lesion size < 1.5 cm)	cT1b cN0 (differentiated type, lesion size < 1.5 cm)	
	Tumour localisation	Middle portion of the stomach	Tumours in the middle portion of the stomach with the distal tumour border at least 4 cm proximal from the pylorus	-
**Pylorus-preserving gastrectomy with D1+ LAD**	Tumour stage	cT1b cN0 tumours that do not meet the criteria for D1 LAD	cT1b cN0 tumours that do not meet the criteria for D1 LAD	cT1 cN0
	Tumour localisation	Middle portion of the stomach	tumours in the middle portion of the stomach with the distal tumour border at least 4 cm proximal from the pylorus	Middle third of the stomach

Some studies suggest that the risk of lymph node metastases in station no. 5 is very low if T1 carcinomas in the middle third of the stomach are involved [38–41]. In contrast, there is evidence of station no. 6 being involved in T1b carcinomas that have a frequency higher than 1% (T1b: 1.8%, [40]).

A meta-analysis of 21 comparative studies, which included 4871 patients with early gastric cancer, demonstrated that long-term survival rates after a pylorus-preserving gastrectomy were comparable to long-term survival rates after a distal gastrectomy [42]. The Korean KLASS04 study is the first multicentre randomised controlled trial to compare the outcome of a pylorus-preserving gastrectomy with the outcome of a distal gastrectomy [37]. The long-term results of this study are still pending.

The goal of pylorus preservation is to reduce dumping syndrome and bile regurgitation, which improves nutritional status and quality of life. However, leaving the pylorus in place carries some risk of delayed gastric emptying. To mitigate the risk, it is necessary to preserve the pyloric branches of the vagal nerve. There are studies that suggest that a longer antral cuff additionally leads to improved gastric emptying [37].

The short-term outcomes of the KLASS 04 study reveal that although gastric outlet obstruction occurs more frequently after pylorus preservation than after distal gastrectomy, the overall postoperative morbidity is not increased [43]. The previously cited meta-analysis of non-randomised studies reveals that pylorus preservation results in a decreased risk of early dumping syndrome, gastritis and bile reflux; there is also stronger recovery of total protein, albumin, haemoglobin and weight compared to distal gastrectomy [42].

## Standard resection procedures

Patients with operable non-early gastric cancer and no evidence of distant metastases require multimodal treatment concepts. These concepts can be curative despite the patients having a high risk of recurrence. Gastrectomy with D2 lymphadenectomy is accepted as the standard surgical procedure. Nevertheless, there is still no consensus on the necessary extent of tumour-free resection margins and the type of reconstruction.

### Total versus subtotal gastrectomy

There is consensus that if there are sufficient tumour-free resection margins, a subtotal gastrectomy can substitute for a total gastrectomy. This is primarily based on three European [44–46] randomised studies and several Asian trials [47–52] revealing that total gastrectomy offers no added oncological value compared to subtotal resection for distal carcinomas. Whether or not subtotal gastrectomy reduces postoperative morbidity is answered in contradictory ways in various studies. A 2016 meta-analysis, which included six randomised studies comparing total to distal gastrectomy for distal gastric cancer, concluded that while subtotal gastrectomy reduces the risk of anastomotic leaks, it has no effect on overall postoperative morbidity [53]. Another meta-analysis investigates total versus subtotal gastrectomy for carcinoma of the distal and middle third of the stomach. Eleven comparative studies that were included revealed a decreased risk of overall postoperative complications, anastomosis leakage, wound complications, peritoneal abscesses and mortality. The stage-specific analysis indicates the same long-term oncological outcome for both procedures [54]. The available evidence cannot be transferred without restrictions to current therapy; the procedures performed in some older trials no longer meet the standards in oncological gastric surgery. Thus, in the study conducted by Robertson et al. [47], only a D1 lymphadenectomy was performed in a subtotal gastrectomy. The total gastrectomy procedure included a D2+ lymphadenectomy with resection of the spleen and pancreatic tail. It should also be noted that randomisation was based on surgeon preference in some studies, which carries a significant risk of bias [46]. The results of a study of the long-term quality of life after a subtotal and a total gastrectomy demonstrate that a subtotal gastrectomy improves the quality of life in many aspects 5 years after surgery. After the 5-year period, most of the differences fade and only less convenient eating restrictions remain in patients treated with total gastrectomy [55]. In summary, subtotal gastrectomy for distal carcinomas appears oncologically equivalent with the possibility of less postoperative morbidity.

### Tumour-free safety margins

The length of the required tumour-free proximal resection margin is considered when determining which patient is suitable for a subtotal gastrectomy. However, there is no consensus on how long a tumour-free resection margin should be. The goal of safety margins is to achieve an R0 resection. The demand for adequate safety margins can be traced to Germany in the 1980s, where the prognosis for the diffuse-type cancer depended on the length of the proximal safety margin. The optimal safety distance was found to be 10 cm in situ, which corresponds to 5 cm on the non-stretched specimen [56]. This finding resulted in recommendations for safety margins of 8 cm for diffuse types and 5 cm for intestinal types. The safety margin for intestinal types was confirmed by a study from seven institutions of the U.S. Gastric Cancer Collaborative; safety distances of 3–5 cm showed a prognostic advantage compared to smaller distances; distances greater than 5 cm did not bring an additional advantage [57]. A noteworthy finding of this study is that there was only a connection between safety margin and survival for stage I. In more advanced stages, other factors were more relevant [57]. Furthermore, a study conducted in Korea demonstrates that although an R1 resection was associated with poorer survival, the exact length of the tumour-free safety margin had no impact on survival [58].

Some studies demonstrate the negative impact of an R1 scenario on overall survival [59, 60]. A study that included patients with proximal gastric cancer from seven centres in the US Gastric Cancer Collaborative questions this; this study found that an R1 margin had no independent impact on recurrence or overall survival [61].

Finally, a meta-analysis of 23 retrospective cohort studies suggests that, apart from gastroesophageal junction carcinomas, positive margins are associated with lower 5-year survival rates [62]. These contradictory results led to recommendations and guidelines that vary by country.

Thus, the Japanese and Chinese guidelines require a proximal margin of at least 3 cm in the case of an expansive growth pattern and at least 5 cm in the case of an infiltrative growth pattern for locally advanced tumours (greater than or equal to T2) that involve the oesophagus. T1 carcinomas are an exception; resection margins of 2 cm may be obtained [4, 63]. In the previous version, the European guidelines required a proximal margin of at least 5 cm for intestinal type and at least 8 cm for diffuse-type tumours [64]. In the current version of the guidelines, the recommendation was adapted to the recommendations of the Asian guidelines by requiring proximal margins of at least 3 cm for tumours with an expansive growth pattern and at least 5 cm for tumours with an infiltrative growth pattern [10]. The changes made to European recommendations are the result of an Italian study that validated the minimal resection margins required by the Japanese guidelines on a Western patient population [65•]. In this study, compliance with the Japanese recommendations for resection margins was independently associated with overall survival. Margin standards determined by Japanese guidelines also had more discriminatory power for survival when compared to the earlier European guidelines [65•].

In the past, for tumours affecting the oesophagus, safety margins of 5–12 cm were required to account for the risk of skip lesions. However, more recent data reveal that safety margins of 2 cm on the fixed specimen or 3 cm in situ are sufficient [13, 66]. However, an intraoperative frozen section examination is obligatory here [4, 10]. In summary, there has been a trend in recent decades towards lower safety margins combined with frozen section examinations, which paves the way for subtotal resections even for tumours in the middle third.

### Extend of lymphadenectomy

There are still questions regarding the optimal extent of lymphadenectomy in gastric cancer. One outcome of the discussion is that D2 lymphadenectomy, which has been a standard procedure in Asia since the 1960s [67], is now also recommended in Western countries. The reason for the initial reluctance to perform D2 lymphadenectomy in Western countries involved two randomised European studies conducted in the Netherlands and the UK that initially failed to show any benefits of D2 lymphadenectomy [68, 69]. The high complication rates, which mostly resulted from the frequently performed pancreatic resection and splenectomy, were also alarming [68]. Thus, pancreatic resection and splenectomy were independently associated with a lower survival rate [69]. According to the 15-year results of the Dutch study, the D2 lymphadenectomy resulted in fewer loco-regional recurrences and decreased disease-related mortality [70]. Both the study from the Netherlands and the study from the UK conclude that avoiding pancreatic resection and splenectomy could lower the morbidity of D2 lymphadenectomy and lead to potential benefits [68, 69]. An Italian phase 2 study on the feasibility of a D2 lymphadenectomy with spleen and pancreas preservation demonstrated that this operation can be performed safely if accompanied by adequate training in specialised centres [71].

The lymph node stations belonging to the D1 and D2 compartments are defined in the Japanese guidelines and depend on the chosen resection. For a total gastrectomy, the D1 compartment includes the peri-gastric lymph nodes (stations 1–7). In the current edition of the guidelines, the D2 compartment also includes the lymph nodes around the hepatic artery, the splenic artery and the celiac trunk (stations 8a, 9, 11p, 11d and 12a). Thus, station 10 (the lymph nodes in the hilum of the spleen) is no longer part of the standard extent of resection for gastric cancer, in contrast to earlier recommendations [4].

For the distal gastrectomy, the para-gastric lymph nodes on the left side of the upper third of the stomach are left for the D1 lymphadenectomy (stations 4sa and 2). The lymphatic tissue on the lesser curvature side, including station 1, must be completely resected as well. The D2 compartment corresponds to that of the total gastrectomy except station number 11d. It should be noted that the distal gastrectomy, which includes at least two-thirds of the stomach in the Japanese guidelines, differs from the four-fifths subtotal gastrectomy performed in Europe [4].

In addition to the D2 compartment, there are individual lymph node stations where it could be beneficial to clear these out under some circumstances.

#### Para-aortic lymph nodes

A routine extension of the D2 lymphadenectomy to include the para-aortic lymph nodes is currently not recommended. This extension offers no oncological advantage, as demonstrated by a randomised study from Japan [72]. In contrast, there is evidence that patients with isolated involvement of the para-aortic lymph nodes in the context of an oligometastatic disease could benefit from it [73, 74].

#### Splenic hilar lymph nodes

According to the current classification, lymph node station 10 is no longer part of the D2 compartment. As a result, routine dissection is not recommended for all tumours [4]. Nevertheless, there is data indicating that proximal gastric carcinomas, and particularly those invading the greater curvature, have a high rate of lymph node metastasis in the splenic hilum [75]. Because dissection of the splenic hilum is very complex, splenectomy was frequently performed in these cases in the past. The added value of the latter was examined for patients with proximal gastric cancer in the Japanese JCOG0110 showing that splenectomy had no oncological benefit but increased postoperative morbidity [76]. Techniques for spleen-preserving dissection of hilar lymph nodes have been investigated in recent years. In addition to patients in whom staging revealed the suspicion of affected lymph nodes in the splenic hilum, a pooled analysis of four prospective trials demonstrated an especially high risk for splenic hilar lymph node metastasis in locally advanced proximal tumours either invading the greater curvature or with a tumour size greater than 5 cm. In this subgroup, the 3-year overall survival rate of the D2+ no. 10 group increased compared to the D2 group [77]. Consequently, the Japanese guideline mentions station 10 dissection as a low-evidence option for proximal carcinoma infiltrating the greater curvature [4].

#### Superior venous mesenteric lymph nodes (no. 14v)

The dissection of the station 14v offering an advantage for patients with distal carcinomas is currently being investigated. Here, lymph node metastases in station no. 6 may be an accurate predictor with a low risk of false-negative results [78]. The involvement of station 14v worsens the prognosis. However, there is evidence that patients with metastasis in lymph node station no. 14v may benefit from the dissection of station 14v [79].

#### Posterior pancreas head lymph nodes (no. 13)

Dissection of station 13 could be considered for distal carcinomas, especially if they infiltrate the duodenum. If they infiltrate the duodenum, the Japanese guidelines do not consider them distant metastases [4]. There is data indicating that involvement of station no. 13 is associated with a poorer prognosis. The question of whether dissection improves the survival of these patients cannot be answered unequivocally with the currently available data [79].

### Reconstruction

The method of reconstruction determines the degree of patient impairment from post-gastrectomy syndromes. An ideal reconstruction provides a reservoir that empties steadily into the small intestine while protecting against bile reflux. In addition, the reconstruction should be simple and less susceptible to technical errors. Various reconstructions have been developed to meet these criteria. However, an international standard for reconstruction after a gastrectomy does not exist.

#### Subtotal gastrectomy

After subtotal gastrectomy, the Roux-en-Y reconstruction is the most commonly performed reconstruction in the world. Another frequently performed alternative is the Billroth II reconstruction. After the distal gastrectomy, the Billroth I reconstruction with preservation of the duodenal passage is primarily performed in Asia as an alternative. A network meta-analysis of randomised controlled trials reveals that the Roux-en-Y reconstruction is superior to the Billroth I and II reconstruction in term of bile reflux and remnant gastritis. In contrast, the Billroth I reconstruction shows more effective emptying of the residual stomach than the Roux-en-Y reconstruction [80]. By comparing Roux-en-Y with Billroth II, a meta-analysis of four RCTs and eight non-randomised studies also demonstrates the advantages of a Roux-en-Y reconstruction, which include less remnant gastritis, reflux esophagitis, dumping symptoms and reflux symptoms [81].

#### Total gastrectomy

As with subtotal gastrectomy, the Roux-en-Y reconstruction is the most commonly performed reconstruction in the world after total gastrectomy. Alternatively, a variety of pouch reconstructions have been developed. The jejunal pouch with Roux-en-Y reconstruction is the most extensively studied pouch. In fact, there is evidence that pouch reconstruction may be superior to reconstruction without a pouch. The source of this evidence is a meta-analysis including 17 randomised controlled trials and eight non-randomised studies [82••]. Different pouch reconstructions were evaluated separately in subgroup analyses, with the jejunal J-pouch being the most common type of reconstruction. Although the pouch application took significantly longer, the more technically complex reconstruction was not accompanied by increased complication rates or a longer hospital stay [82••]. Pouch reconstruction reduced the risk of developing dumping syndrome. This difference existed both after 3–6 months and after 12–24 months. Other benefits of pouch reconstruction included less esophagitis, less heartburn, less dumping syndrome and fewer problems with feeding. These effects still existed 1–2 years postoperatively. The nutritional status was also better after that period [82••]. An additional Chinese cohort study underlines the advantage of pouch reconstruction by demonstrating superiority in terms of the quality of the patient’s life and dumping syndromes [83].

## Minimally invasive approaches

### Laparoscopic

Minimally invasive procedures are growing in popularity in the field of oncological gastric surgery. The feasibility and oncological safety of laparoscopic gastrectomy have been demonstrated in RCTs conducted in Asia. Thus, the Korean KLASS01 study revealed lower rates of wound complications when using the laparoscopic technique in distal gastrectomy for stage I carcinomas. The long-term oncological outcome was equal when the technique was compared to open distal gastrectomy [84, 85]. The Japanese JCOG0912 trial also supported the non-inferiority of the laparoscopic approach compared to the open distal gastrectomy for clinical stage I gastric cancer relapse-free survival [86•]. The short-term results of the study indicate a decreased time to first flatus, lower intraoperative blood loss and less need for pain medication in the laparoscopic approach [87]. The Chinese CLASS-02 study examines the technically complex total gastrectomy for stage I carcinoma. In this study, the laparoscopic method was equally safe in terms of short-term morbidity and mortality when compared to the open total gastrectomy [88].

For locally advanced carcinomas, there have long been concerns as to whether a D2 lymphadenectomy can be properly performed radically and laparoscopically. There is strong evidence from RCTs that laparoscopic gastrectomy can also be safely performed in locally advanced carcinomas [89]. The Korean KLASS-02 [90•] and the Chinese CLASS-01 [91•] studies indicate that laparoscopic surgery is equal to the open procedure. The Dutch LOGICA study complements the existing data and also shows comparable results for postoperative complications and the 1-year survival rates [92•]. A recent meta-analysis comparing open versus laparoscopic for locally advanced tumours, which included 12 RCTs, reveals advantages of the laparoscopic approach in terms of intraoperative blood loss and length of hospital stay [93]. The number of resected lymph nodes was lower in the laparoscopic group. However, since this is an average difference of one lymph node, the biological significance must be questioned. The equivalent long-term survival rate for both groups also indicates that both procedures are oncologically equal. Another meta-analysis includes 17 RCTs with early and locally advanced cancer; findings include advantages for the laparoscopic group regarding intraoperative blood loss, the need for analgesics, the time to the first flatus, the length of the hospital stay, oral intake and the overall complication rate [94]. Thus, this meta-analysis provides first evidence that laparoscopic gastrectomy could even be superior to open surgery with regard to the early postoperative course. However, there is still a need for further clarification on some points. The anastomosis is one of the central steps in the reconstruction. Due to the technical difficulties of intra-corporeal anastomoses, most of the above studies use the specimen retrieval incision for an extra-corporeal reconstruction. In obese patients in particular, it may be necessary to significantly widen the incision to create the anastomosis. In addition, an extra-corporeal reconstruction requires a specimen retrieval incision in the upper abdomen. This type of incision in the abdomen has disadvantages in terms of wound pain and the frequency of incisional hernias. In this regard, intra-corporeal reconstructions with specimen retrieval via a Pfannenstiel incision could offer advantages.

Another problem is that distal resection is predominantly performed, according to the available literature. In Western countries, total gastrectomy predominates, which is a much more demanding form of reconstruction. The technical demands increase even further when a pouch reconstruction is carried out, especially when it is carried out intra-corporeally. It is currently unclear whether the advantages of laparoscopy are even greater in this case or whether the difficulty of laparoscopic reconstruction leads to higher complication rates.

### Robotic

Robotic surgery has become more common in surgical procedures over the past few decades. The advantages of robotics include three-dimensional visualisation, higher magnification, easier instrument movement and better ergonomics. Robotics also provides promising advantages for oncological gastric surgery. However, the evidence for this promise is currently weak. A Chinese RCT examines the difference between robotic and laparoscopic distal gastrectomy in patients with cT1-4a and N0/+ tumours. The advantages of robotics being used in the RCT included lower morbidity rate, faster recovery, milder inflammatory responses and improved lymphadenectomy [95]. These results are consistent with those of a meta-analysis of observational studies demonstrating the benefits of the robotic procedure for intraoperative blood loss, number of lymph nodes harvested and length of hospital stay [96]. Furthermore, a Japanese randomised trial examining the superiority of robotic to laparoscopic gastrectomy does not meet the end point of reducing intra-abdominal infectious complications with robotic gastrectomy. Nevertheless, in this study, the robotic procedure has fewer postoperative complications of grade II or higher [97]. Thus, the scant evidence available suggests that robotic gastrectomy is advantageous, although the usefulness of these preliminary results may vary.

## Surgery for oligometastatic disease

There is a profuse amount of speculation regarding surgery for limited metastatic gastric cancer. In addition to meta-analyses that contain non-randomised studies, there are currently few randomised studies.

The German study conducted by Al-Batran et al. prospectively divided patients into groups with different degrees of metastasis. The group with only limited metastasis received chemotherapy and surgery and displayed higher survival rates than the extensively metastatic patient group, in which surgery could not be considered [98].

A study conducted in the UK demonstrated that patients with synchronous liver metastases from gastric cancer can benefit in terms of survival from a simultaneous liver metastasis resection without postoperative morbidity being increased [99]. Additionally, analysis of patients with metastatic gastric cancer from the SEER database revealed that resection of the primary tumour and metastases was an independent prognostic factor for survival [100]. The challenge is to define which criteria the metastases should meet for the patients to benefit from the resection. A meta-analysis of such factors yielded the following criteria: fewer than three metastases with a size of less than 5 cm, which are only in one lobe of the liver [101].

The Asian REGATTA study is a prospective, randomised study that compared palliative chemotherapy with gastrectomy followed by chemotherapy in patients with gastric cancer with single-site metastasis. This study failed to demonstrate a survival benefit of resection [102]. However, the metastases were not resected here; only the primary tumour was resected. In addition, there was no preoperative chemotherapy. Thus, the results cannot be transferred to the current discussion.

Two RCTs are currently recruiting participants for the investigation of surgical therapy in oligometastasis. The FLOT5 study tests the hypothesis that a selected subgroup with limited metastatic disease after chemotherapy would benefit from resection of the primary tumour and metastases. The second RCT is the French SURGIGAST study comparing the continuation of chemotherapy versus surgical removal of the primary tumour and the metastatic site in oligometastatic gastric cancer.

Conditions in which patients may benefit from resection of the tumour and metastases after chemotherapy are R0 resection, stable condition, single metastases, absence of peritoneal carcinoma or no further tumour manifestations and a significant response of the tumour to systemic chemotherapy. The resection of solitary metachronous liver metastases and solitary metachronous ovarian metastases (Krukenberg tumours) may also be appropriate for patients under similar conditions. A relevant prerequisite for resection should be the possibility of R0 resection of the primary tumour and metastases in the synchronous situation or of the metastases in the metachronous situation, as well as previous chemotherapy.

In summary, there are indications that some patient populations could benefit from surgical therapy after chemotherapy in the oligometastatic context. Due to the lack of evidence regarding this course of action, including such patients in ongoing studies is feasible.
